# Myenteric Denervation of the Gut with Benzalkonium Chloride: A Review of Forty Years of an Experimental Model

**DOI:** 10.1155/2019/3562492

**Published:** 2019-02-03

**Authors:** Sérgio Britto Garcia, Stefânia Bovo Minto, Isabela de Souza Marques, Vinicius Kannen

**Affiliations:** ^1^Department of Pathology and Legal Medicine, Faculty of Medicine of Ribeirão Preto, University of Sao Paulo, Av. Bandeirantes 3900, Ribeirão Preto 049-900, Brazil; ^2^Department of Toxicology, Bromatology, and Clinical Analysis, University of Sao Paulo, Av. Bandeirantes 3900, 14, Ribeirão Preto 049-900, Brazil

## Abstract

Experimental denervation of organs plays a key role in understanding the functional aspects of the normal innervation as well as the diseases related to them. In 1978 the experimental model of myenteric denervation of the rat gut by serosal application of benzalkonium chloride (BAC) was proposed. BAC is a positively charged surface-active alkylamine and is a powerful cationic detergent, which destroys bacteria after ionic attraction and for this reason is largely used as a surgical antiseptic. Since its initial report, the BAC-induced myenteric denervation model has been used to study many functional and pathological aspects of the enteric nervous system. So far this is the only pure method of myenteric denervation available for research in this area. Promising reports in the literature have shed light on the possibilities for the development of new uses of the BAC-denervation experimental model as a therapeutic tool in some pathological situations. This review aims to shed light on the main historical and recent findings provided by this experimental model.

## 1. Innervation and Pathology of the Gut

The gut is extrinsically innervated by the sympathetic and parasympathetic divisions of the autonomic nervous system and intrinsically innervated by the enteric nervous system (ENS). The ENS is a collection of millions of neurons that controls or is involved in virtually all the gut functions [1]. It acts largely independently from the central nervous system, as for example the well documented presence of the intestinal peristalsis “in vitro” [2]. The ENS has sensorial receptors and afferents, as well as motor and integrative neurons. Such complexity and independent functions have led to the proposal to classify the ENS as a third division of the autonomic nervous system, along with the sympathetic and parasympathetic divisions [3]. The ENS comprises the myenteric plexus (also known as the Auerbach plexus), located between the longitudinal and circular muscle layers of the gut wall, and the submucosal plexus (also known as the Meissner plexus).

Changes in the ENS, due to illness or age, culminate in loss of gastrointestinal functions. In the primary enteric neuropathies the ENS is considered to be the primary target of the disease process. On the other hand, in the secondary neuropathies the ENS is affected as part or consequence of a gut or a systemic disease. A review has discussed the main clinical primary enteric neuropathies: achalasia, gastroparesis, intestinal pseudoobstruction, and chronic constipation [4]. Interestingly, these neuropathies can be experimentally simulated by the BAC-induced myenteric denervation, as we will further discuss in the next sections of this text.

## 2. Experimental Models of Gut Denervation

Experimental denervation of organs plays a key role in much, if not most of the understanding of the functional aspects of the normal innervation as well as the diseases related to it. Since the fifties surgical experimental models of gut extrinsic denervation have been developed and validated to study intestinal physiology and pathology, as for example, the studies with vagotomy and splanchnicectomy [5]. Pharmacological blockade has also been used to achieve either the sympathetic or the parasympathetic denervation of the autonomic nervous system [6]. Nevertheless, despite their usefulness for various scientific purposes, both surgical and pharmacological approaches for the gut denervation present important limitations. First, apart from some minor reactive changes, the extrinsic surgical denervation of the gut does not abolish the main functions of the ENS [7] and reinnervation or adaptation frequently occurs after the surgical procedure [8]. Second, the pharmacological blockades not only are nonspecific for the ENS or myenteric plexus, but also cause profound systemic consequences that may cause significant bias in the studies of the ENS, as observed after either sympathetic or parasympathetic blockade with guanethidine or atropine, respectively [9, 10].

## 3. Intrinsic Denervation of the Gut with Benzalkonium Chloride: Historical and Mechanistic Backgrounds

Benzalkonium salts comprise a group of positively charged surface-active alkylamine biocides with the general formula alkyldimethylbenzylammonium chloride (BAC) or bromide [11]. For decades BAC has been largely used as an ophthalmic and nasal antiseptic and as a skin disinfectant in surgery [12]. BAC is a powerful cationic detergent that destroys bacteria and also mammal tissues due to its ability to disrupt cell membranes in general, as confirmed with the observation by scanning electron microscope, of extensive cell membranes lesions in the corneas of rabbits treated with BAC [13].

Interestingly, in 1978, Sato and colleagues [14] found that the administration of BAC topically applied on the intestinal serous layer leads to a specific and irreversible injury of the myenteric (intramural) nerve cells. In fact, these authors had observed that the BAC serosal application also resulted in degenerative lesions of the other cells of the intestinal wall, but these lesions disappeared in a few weeks after the application of this surfactant, with total recovery of the intestinal wall, apart from the myenteric neurons [14]. In sequence, Sakata et al. (1979) studied electron-microscopically the effects of the BAC serosal application on the intestinal wall and proposed that a lower recovering ability of the nerve elements might be responsible for the specificity of neural lesions in this experimental model [15].

Nevertheless, so far, the mechanisms for the specificity of the neuron lesions after BAC exposure remain not well known. It was further observed that higher concentrations of BAC caused a generalized tissue damage including disruption of the smooth muscle, lymphocytic infiltration, intestinal perforation and death [16]. The time sequence of the BAC-induced effects on the muscle layers was also determined. Within 24 h the total destruction of the longitudinal muscle and partial destruction of the circular muscle were evident and the myenteric plexus was necrotic. By 5 days after treatment, both muscle layers had regenerated to their original states and the myenteric plexus was totally absent [17]. Possibly the submucous neurons are not affected by BAC application because they are more distant than the myenteric neurons from the serosa. This hypothesis is supported by the observation that BAC only causes minimal lesions on the internal (circular) muscle layer of the intestinal wall [17].

Another mechanistic study showed widespread apoptosis in the myenteric plexus 3 days after the BAC treatment and a loss of more than 90% of myenteric neurons one week after the treatment. A marked increase in populations of T cells, B cells, and macrophage-like cells was also observed. Cyclosporine partially reversed the inflammatory cells infiltration and delayed the neuronal loss, suggesting that the immune response may actively contribute to tissue destruction in this experimental model [18]. It has been observed in conjunctival cells that the mode of BAC-induced cell death is dose- dependent. BAC treatment leads to necrosis at high concentrations and apoptosis at low concentrations [19]. Possibly similar situation occurs in the gut, but more studies are necessary to clarify the detailed mechanisms of BAC neuronal destruction. Finally, a recent study has shown in the rat corneas that the levels of total *β*-catenin were significantly upregulated 8 h after the topical administration of BAC, suggesting that the cytotoxicity of BAC may be also mediated through the modulation of the Wnt pathway [20].

## 4. Effects of Myenteric Denervation by BAC on the Organs of the Digestive Tract

### 4.1. Esophagus

In humans, achalasia of the esophagus is associated with abnormalities of the myenteric plexus. The application of BAC on the lower esophagus of rats provided an experimental model of achalasia, with distal narrowing, proximal dilatation and decreased food intake [21]. Other study further characterized this achalasia experimental model through an injection of BAC circumferentially into the lower esophagus and the esophagogastric junction (EGJ) in opossums, resulting in manometrically higher pressures and loss of nitric oxide expressing inhibitory myenteric neurons with failure of relaxation of the sphincter by electrical stimulation [22]. We have obtained similar findings in rats with the lower esophagus denervated by BAC, which was associated with the formation of megaesophagus with a considerable increase in thickness of the muscle layers [23].

It must be mentioned that EGJ distensibility is an important factor in the management of human achalasia [24] and a subgroup of patients presents manometrically normal EGJ relaxation but has an impaired EGJ distensibility [25]. Unfortunately, the available experimental studies did not evaluate specifically the denervation of the EGJ. All of them included the denervation of the lower esophagus as well, which is a confounding factor in the interpretation of the results. Thus, experimental studies with injection of BAC circumferentially only into the EGJ are warrant. Interestingly, the denervation with botulinum toxin has also been used for the treatment of human achalasia with reasonable but only transient responses [26]. It may be hypothesized that reinnervation of the lower esophageal sphincter could be related to the transitory results of this procedure, since the botulinum toxin is degraded along time [27]. Thus, it is possible to hypothesize that the application of BAC could eventually be more successful than the botulinum toxin for the treatment of achalasia in the long term, since it causes a permanent loss of neurons. Nevertheless, limitations of human studies with BAC will be further discussed in this review

### 4.2. Stomach

The first study of the BAC serosal application in the stomach, performed on domestic turkeys, showed that this procedure resulted in the loss of motility with hypertrophy of the caudoventral muscle of the stomach and a decrease in the number and size of myenteric neurons [28]. In another study in turkeys, the denervation of the isthmus between the glandular (GS) and muscular stomach (MS) abolished the GS contractions and reduced the frequency of the MS and duodenal contractions by 50%. Pyloric denervation did not affect the frequency of the GS or MS contractions but abolished the duodenal contractions, suggesting that a driving pacemaker for the gastroduodenal cycle is located in the isthmus and that the myenteric plexus is essential for the conduction from the pacemaker to the GS and to the duodenum [29]. Ablation of the nerves beneath the medial or lateral side of the gastric isthmus by BAC indicated that initiation and regulation of the muscle involved in motility acts via nerves encircling the isthmus [30].

Studies in rats showed that duodenal contractions occurred spontaneously in the coordination with antral contractions in normal stomach preparations. Interestingly, when the antroduodenal junctional zone was pretreated with BAC, the augmented duodenal contractions did not occur spontaneously, suggesting that myenteric neurons mediate antroduodenal coordinated contractions [31]. The serosal BAC application on the rat antrum or corpus caused a disturbance in the emptying of both liquids and solids, as well as a dose-related specific loss of gastrin releasing peptide, substance P, and vasoactive intestinal polypeptide immunoreactivities [32]. The antral denervation with BAC suggested that luminal nutrient releases gastrin in the rat via activation of the antral neurons secreting gastrin-releasing peptide and that the antral innervation normally inhibits G-cell responses to nonnutrient distension of the stomach [33].

The BAC experimental model also showed that not only are functional aspects of the stomach affected by myenteric denervation, but also they exert profound effects on the epithelial sheet, with an increase in the mucosal area of all gastric portions (hyperplasia), as well as an increase in the gastrin-G cell and somatostatin-D cell populations. Interestingly, these changes did not occur when denervation was combined with pyloroplasty, indicating that, after denervation, gastric distension and stasis are the major stimuli for hyperplasia [34]. The role of gastric food stasis as a major factor triggering morphological alterations of the gastric epithelium has been reinforced by the observation that the BAC-denervation of the rat stomach increases gastric emptying time, acid, and the gastrin secretion together with the increase of the size of the mucosa [35].

### 4.3. Small Intestine

Five years after the publication of the BAC-induced colonic denervation, the effects of BAC application in the small intestine of rats were well described by Fox et al. (1983). These authors have observed a significant reduction in the number of ganglion cells in the myenteric plexus “as early as 5 days after BAC treatment”, which persisted over time [16]. The most important findings made with BAC application in the small intestine are related to discoveries of a variety of myenteric plexus functions. Initially, it has been observed that the BAC-induced ablation of the myenteric neurons in the rat jejunum disrupts the basic electric rhythm but not the migrating myoelectric complex propagation, suggesting that the myenteric neurons play a modulatory role in the generation and propagation of this rhythm, whereas humoral factors or the submucosal neurons may be more important in the control of the migrating myoelectric complex [36]. The BAC experimental model also showed that the motor neurons innervating the rat jejunal longitudinal muscle are located in the myenteric plexus, because the muscle responses are lost despite the presence of an intact submucosal plexus [37]. Furthermore, a set of experiments with pharmacological blockers of the autonomic system in rats showed that adrenergic receptor agonists produced a concentration-dependent relaxation of longitudinal muscle of the rat jejunum, but myenteric denervation with BAC caused an impairment of the relaxation, indicating that myenteric neurons play a role in the sympathetic modulation of the intestinal wall [37]. The myenteric neurons were also observed to influence the mechanical responses of isolated rat jejunal longitudinal muscles produced by acetylcholine, 5-hydroxytryptamine, cholecystokinin octapeptide, norepinephrine, and vasoactive intestinal peptide [38]. Other studies have confirmed that the myenteric denervation importantly reduced the cholinergic responsiveness of intestinal smooth muscle [39], impaired the intestinal motility [40], and influenced the neurotransmitter levels and myoelectrical activity [41].

The loss of myenteric neurons is associated with marked alternations in intestinal morphology, leading not only to visceral dilation but also to the thickening of the smooth muscle layers, as seen in the jejunum and ileum [16, 42–44]. This increase in thickness of both the longitudinal and circular smooth muscle layers is thought to be primarily due to an increase in the number of smooth muscle cells (hyperplasia), since little cellular hypertrophy was previously observed [16]. Myenteric denervation leads to the replacement of the loosely arranged reticulin fibers surrounding the smooth muscle cells by fibrous tissue (with typical collagen fiber), which may alter the biomechanical function resulting in the impairment of muscular contraction [45]. It has been proposed that the muscles may thicken because motility is abnormal in the myenteric denervated segment [36, 40] or in response to the abnormal innervations of the tissue, since changes in the levels of various neurotransmitter occur in response to chemical ablation of myenteric neurons [41]. We consider that it is likely that both mechanisms are responsible for the thickening of the muscle and mucosa intestinal layers.

Myenteric plexus also regulates mucosal morphology and functions. The BAC-induced denervation caused increased villus height and crypt depth [43, 44, 46], as well as increased crypt cell proliferation rates [47, 48]. In suckling and weanling rats, the myenteric ablation was followed by a sharp increase on cell proliferation and migration in the ileal epithelium in the first 15 days of treatment with a subsequent recovery of the intestinal mucosa homeostasis later on [49]. The BAC myenteric denervation provided information relating myenteric neurons with secretion and absorption functions of the intestinal mucosa. The BAC treatment eliminated the ability of cholera toxin to induce intestinal secretion, which suggests that the secretory reflexes activated by cholera toxin are probably conveyed via the myenteric plexus [50] and ablation of the jejunal myenteric plexuses by treatment with BAC reduces alanine absorption [51].

Finally, the BAC experimental model has also provided new insights on the intestinal physiology. In a study of rats surgically prepared with Thiry-Vella intestinal loop, the “in vivo” distension of the normal loops reduced fluid intake and produced signs of food aversivity whereas these effects were absent in the BAC-treated animals, showing that this experimental model can be used to study the mechanisms underlying the effects of the enteral stimuli on the feeding behavior [52]. This experimental model also showed a role played by the myenteric plexus on intestinal inflammation. Interestingly, the BAC-denervation abolished the protective effects provided by both the ketamine and the vagal nerve stimulation against ischemia/reperfusion injury [53, 54].

### 4.4. Colon

The first experiment with the myenteric denervation by BAC was performed in the colon of rats (Sato et al., 1978) and the authors observed that viscerae become “dilated as in megacolon” [14]. Other studies reported a close resemblance between the BAC-induced aganglionic colon and the more proximal segments of Hirschsprung's disease megacolon [55–59]. Recently, a new method has been described to achieve an even more accurate animal model of the Hirschsprung disease, by using colonoscopy to inject BAC, and to produce ablation of the myenteric nervous system in the distal colon and rectum in mice [60].

The main morphological alteration described in the BAC- induced experimental megacolon is an increased thickness of the colon wall, which is due to muscle hypertrophy and mucosal hyperplasia [56]. The denervated colonic mucosa presents an increase in the crypt cell population, crypt cell production per crypt, and a decrease in cell cycle time [56]. A functional study with anorectal manometry in mice with aganglionic rectums showed the absence of slow waves and high resting pressure similar to the congenital aganglionic rectums. This finding suggests that hypertrophic nerves usually found in human congenital aganglionosis may not be necessary to produce the anorectal spasticity [57].

## 5. New Research Directions and the Potential Use of Myenteric Denervation with BAC as a Therapeutic Tool for Some Gut Diseases

The BAC experimental model can be used as a tool to develop innovative approaches for the treatment of diseases related to myenteric neurons absence or damage. Interestingly, the transplantation of enteric neural stem cells into the mice aganglionic colon induced by BAC was performed. After this procedure it has been observed that the expression of the neural and glial markers was increased and the colonic motility was improved, which may provide a potential novel therapy for the treatment of Hirschsprung's disease [61]. Other study has shown that the treatment with a selective agonist of the estrogen receptor *β* increased the recovery of neurons after damage by BAC application [62]. Both studies demonstrate the potential of the BAC experimental model for neuroplasticity research and the testing of new treatment strategies for ENS diseases.

On the other hand, scattered reports in the literature for decades have been suggesting that denervation with BAC could provide new therapeutic approaches in some gut diseases. In the first of these reports, the denervation with BAC improved survival and weight gain and increased transit time and d-xylose absorption in rats following 80% small bowel resection [63]. These findings were confirmed by other studies that have shown that BAC-denervation improved clinical conditions of the animals and produced a marked increase of the remaining intestinal absorptive surface in the short-bowel syndrome [64–66].

In the stomach, the BAC-denervation procedure has been proposed as a new method to perform parietal cell vagotomy in substitution of the traditional operative procedure, which is an accepted therapy for peptic ulcers that are refractory to medical management [67]. Furthermore, a comparative study of efficacy of several techniques in regard to long-term effects on acid secretion, ulcer prophylaxis, and permanent vagal denervation in a rat model has shown that the BAC chemoneurolytic and laser methods were most effective for decreasing the size of acid-secreting areas [68, 69].

Another field of investigation has been provided by the evidence that myenteric denervation with BAC exerts a protective role against colon cancer [70, 71] and stomach cancer [72]. This research line, despite being incipient, may be useful on the investigation of the poorly known relationship between cancer and the autonomic nervous system.

Nevertheless, there are significant limitations for the potential therapeutic use of BAC in clinical situations. First, the application of BAC in the gut serosa causes transitory peritonitis that is well tolerated in rodents, but certainly could not be acceptable in humans. Second, even if the BAC could be applied in the intestinal wall trough an endoscopic route, as it has been used in some experimental studies mentioned in this text, it probably would be very difficult to achieve a precise amount of denervation in one given clinical situation. In that case, it is reasonable to hypothesize that an insufficient or an excessive degree of neuron loss could cause insufficient results or undesirable side effects, respectively. Third, considering that the BAC-denervation impairs the visceral peristaltism, possible problems associated with pseudoobstruction of the viscera in the long term may be expected. In view of these considerations, despite being promising, the future directions for the research towards the use of BAC as a therapeutic tool must take into account the fact that major technical improvements must be achieved related to the efficacy, precision, and safety of the procedure prior any eventual use in clinical settings.

It should be mentioned that all the above limitations regarding the clinical use of BAC are present when it is used as a compound for disease modelling in experimental settings as well. Thus, both side effects and a lack of precision on the amount of denervation must be taken into account in the design of experiments. Finally, the possibility of partial recovery of innervation along time due to neuroplasticity phenomena should not be ruled out.

## 6. Conclusions

The gut myenteric denervation with BAC has been shown to be a simple, nonexpensive, and reliable experimental model. It has been providing interesting information about the functional and pathological aspects of the enteric nervous system during the last forty years. New research lines are likely to emerge from the use of this experimental model, including some potential therapeutic applications. Nevertheless, important technical limitations must be taken into account in experimental settings and must necessarily be overcome before any clinical use.
